# Tuberculosis Infection among Non–US-Born Persons and Persons ≥60 Years of Age, United States, 2019–2020

**DOI:** 10.3201/eid2907.230324

**Published:** 2023-07

**Authors:** Rachel Woodruff, Roque Miramontes

**Affiliations:** Centers for Disease Control and Prevention, Atlanta, Georgia, USA

**Keywords:** tuberculosis and other mycobacteria, latent tuberculosis infection, LTBI, tuberculosis disease, non–US-born, NHANES, bacteria, respiratory infections, United States

## Abstract

We examined tuberculosis (TB) infection results for the United States from the 2019–2020 National Health and Nutrition Examination Survey. Over this period, 10% of non–US-born persons and 7% of those >60 years of age tested positive for TB infection. These results provide up-to-date information on TB infection among study subpopulations.

Each year, the Centers for Disease Control and Prevention (CDC) collects demographic and clinical data for tuberculosis (TB) disease cases reported from all 50 states and the District of Columbia (*1*). Studies that examined the completeness and accuracy of the national TB surveillance system found that this reporting consistently and accurately captured close to 100% of all diagnosed cases and is therefore highly representative of the burden of TB disease in the United States (*2**–**4*).

The precursor to TB disease, latent TB infection (LTBI), is not a reportable condition in most US states (*5*), so the number of infected persons is not well understood. Beginning in 1970, CDC funded a TB component of several National Health and Nutrition Examination Surveys (NHANES) that provide the primary source of national data on LTBI in the US population (*6**–**8*). Most recently, CDC provided funding for the 2019–2020 NHANES survey to examine LTBI among non–US-born persons >6 years of age and all persons >60 years of age. The 2019–2020 NHANES protocol was reviewed and approved by the NCHS Ethics Review Board (https://www.cdc.gov/nchs/nhanes/irba98.htm).

NHANES is a series of sequentially run cross-sectional surveys that assess the health of the civilian, noninstitutionalized US population (*9*). To obtain a nationally representative sample, NHANES employs a complex, stratified, multistage probability cluster sampling design (*9*). The sample for the NHANES 2019–2020 survey cycle was intended to be nationally representative and included 30 primary sampling units (*10*). However, 2019–2020 NHANES data collection was suspended in mid-March 2020 because of the COVID-19 pandemic. At that time, data collection had been completed in only 18 of 30 primary sampling units. Because data collection was incomplete, calculating survey weights for that partial cycle was not possible, and the sample was therefore not nationally representative. As a result, only an unweighted partial dataset from the Centers for Disease Control and Prevention’s National Center for Health Statistics (NCHS) Research Data Center was available for analysis. 

## The Study 

Non–US-born 2019–2020 NHANES study participants >6 years of age and all study participants >60 years of age were eligible to be tested for TB infection using an interferon-gamma release assay (IGRA), QuantiFERON-TB Gold Plus (QIAGEN, https://www.qiagen.com). We selected those specific subpopulations of persons because of their high IGRA test positivity in previous studies (*8**,**11*), the potential for complications of TB disease in the >60 age group, and the high risk of LTBI progressing to TB disease among non–US-born persons (*11*). We excluded persons in those subpopulations who did not undergo phlebotomy as part of the routine NHANES exam. Data for coexisting conditions were not available in the 2019–2020 NHANES dataset (*12*). All IGRA test results were provided by a single Clinical Laboratory Improvement Act–certified laboratory. Qualitative results were reported for each IGRA test result based on criteria provided by the manufacturer. We used R statistical software (The R Foundation for Statistical Computing, https://www.R-project.org) to obtain frequencies and percentages of eligible NHANES participants with positive TB test results by demographic characteristics. Because of NCHS confidentiality policy, Research Data Center results were suppressed for all categories and subcategories that had <5 responses (*13*). 

In the 2019–2020 NHANES study, 837 non–US-born participants >6 years of age were tested for TB infection, of whom 85 (10%) had positive TB test results (Table). Among non–US-born study participants who tested positive for TB, 51% (43/85) identified as non-Hispanic Asian and 35% (30) as Hispanic; 79% (67) had been in the United States for >5 years. In comparison, among all non–US-born study participants (irrespective of TB test results), 35% (290) identified as non-Hispanic Asian and 43% (356) as Hispanic. 

**Table:** Characteristics of non–US-born study participants >6 y of age and all study participants >60 years of age who were eligible for TB testing, United States, 2019–2020*

Characteristic	Non–US-born persons ≥6 y, no. (%)			All persons ≥60 y, no. (%)	
	Positive TB test	All		Positive TB test	All
All participants	85 (100.0)	837 (100.0)		81 (100.0)	1,123 (100.0)
Sex					
M	42 (49.4)	408 (48.7)		43 (53.1)	582 (51.8)
F	43 (50.6)	429 (51.3)		38 (46.9)	541 (48.2)
Age group, y					
6–14	NA	65 (7.8)		NA	NA
15–24	NA	72 (8.6)		NA	NA
25–44	28 (32.9)	278 (33.2)		NA	NA
45–59	30 (35.3)	244 (29.2)		NA	NA
≥60	24 (28.2)	178 (21.3)		81 (100.0)	1,123 (100.0)
Race/ethnicity					
Non-Hispanic White	NA	55 (6.6)		17 (21.0)	495 (44.1)
Non-Hispanic Black/African American	NA	113 (13.5)		35 (43.2)	357 (31.8)
Hispanic/Mexican American	30 (35.3)	356 (42.5)		19 (23.5)	177 (15.8)
Non-Hispanic Asian	43 (50.6)	290 (34.6)		NA	55 (4.9)
Other	NA	23 (2.7)		NA	39 (3.5)
Poverty					
At or above poverty level	60 (70.6)	535 (63.9)		51 (63.0)	791 (70.4)
Below poverty level	NA	150 (17.9)		14 (17.3)	162 (14.4)
Unknown	NA	NA		16 (19.8)	170 (15.1)
Education level					
No high school diploma	32 (37.6)	312 (37.3)		26 (32.1)	230 (20.5)
High school graduate	17 (20.0)	133 (15.9)		23 (28.4)	300 (26.7)
Beyond high school	36 (42.4)	391 (46.7)		32 (39.5)	592 (52.7)
Unknown	NA	NA		0	NA
Birthplace					
Within United States	0	0		57 (70.4)	945 (84.1)
Outside United States	85 (100.0)	837 (100.0)		24 (29.6)	178 (15.9)
Time in the United States, y†					
<1	NA	29 (3.5)		NA	NA
1–4	NA	118 (14.1)		NA	6 (33.7)
≥5	67 (78.8)	653 (78.0)		20 (83.3)	162 (91.0)
Unknown	NA	37 (4.4)		NA	6 (33.7)

*NA, not available (data not collected for this subpopulation or not reported because cell size <5). †Percentages were calculated using the numbers of persons born outside the United States as the denominator.

Among the NHANES study participants who were >60 years of age (including both US-born and non–US-born persons), 1,123 were tested for TB, among whom 81 (7%) had positive TB test results (Table). Among those who tested positive for TB, 43% (35) identified as non-Hispanic Black. Among study participants >60 years of age, those with TB-positive test results were less likely than the overall total to identify as non-Hispanic White (21% vs. 44%), more likely to be living in poverty (17.3% vs. 14.4%), less likely to have at least a high school diploma (32.1% vs. 20.5%), and more likely to have been born outside the United States (29.6% vs. 15.9%). 

All TB-positive non–US-born NHANES study participants >6 years of age and TB-positive participants >60 years of age were more likely to identify within racial and ethnic minority groups, not have at least a high school diploma, and be living in poverty than overall study participants in each of those subpopulations. In addition, participants >60 years of age who tested positive for TB were more likely to have been born outside the United States than overall study participants >60 years of age. Although not directly comparable across studies, data from our study about demographic differences between TB-positive and overall study participants for both subpopulations were consistent with previous research (*8**,**11*). 

Comparing data between the 2019–2020 and previous NHANES studies was also limited by a change in the way participants were classified into US-born or non–US-born categories. For the 2019–2020 survey cycle, NHANES study participants were considered US-born if born in a state or the District of Columbia within the United States or in a US-affiliated territory; for the 2011–2012 study, participants born in a US-affiliated territory were considered non–US-born. Because country of birth is one of the most critical stratification factors for examining characteristics of LTBI, defining country of birth consistently will be essential in future NHANES survey cycles that include TB testing. 

## Conclusions

Although the 2019–2020 NHANES TB test results were not representative of the US population, study data still indicate that TB infection remains a critical health screening consideration among non–US-born persons and persons >60 years of age. Therefore, persons in these subpopulations should be prioritized for TB testing and, if TB infection is diagnosed, treated with an appropriate regimen to prevent progression to active TB disease (*14*,*15*). Furthermore, the likely disruption of testing and treatment of TB infection that occurred because of the COVID pandemic suggests we still have much to do if we are to eliminate TB in the United States (*1*). 
